# Visualization of Endosomal Escape of Intracellularly Delivered Protein With Unexpected Photochemical Internalization of Trypan Blue

**DOI:** 10.1002/advs.202516456

**Published:** 2026-04-10

**Authors:** Zhongqi Yao, Zhan Shi, Peng Hu, Changping Wang, Hui Wang

**Affiliations:** ^1^ School of Emergent Soft Matter South China University of Technology Guangzhou China; ^2^ Department of Orthopedics Shanghai General Hospital Shanghai Jiao Tong University School of Medicine Shanghai China

**Keywords:** endosomal escape, intracellular protein delivery, photochemical internalization, polymer carrier, protein release

## Abstract

Intracellular protein delivery holds great potential for biomedicine and biotechnology, but real‐time monitoring of protein release remains challenging. Here, we introduce trypan blue (TB) as a multifunctional agent for both tracking and enhancing protein delivery. TB forms a supramolecular complex with GFP, quenching its fluorescence, and further assembles with a cationic guanidine‐rich polymer into a stable nanostructure. Upon 590 nm light irradiation, TB acts as a photosensitizer, triggering photochemical internalization (PCI) that promotes endosomal escape and protein release into the cytosol, accompanied by recovery of GFP fluorescence. This TB‐mediated PCI not only enhances delivery efficiency but also allows real‐time visualization of protein binding and release. We demonstrate the utility of this system through in vitro and in vivo delivery of functional proteins, including the cytotoxic protein saporin, which exhibits light‐activated antitumor activity. Our work highlights TB as a simple yet powerful tool for advancing intracellular protein delivery and tracking.

## Graphical abstract

TB, a conventional cell viability dye, acts as both a fluorescence modulator (quenching GFP fluorescence upon protein binding and recovering it upon release) and a photosensitizer that triggers endosomal escape via photochemical internalization (PCI) under 590 nm light. This dual functionality enables real‐time visualization of protein dynamics while enhancing delivery efficiency.

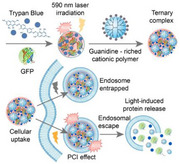

## 1 Introduction

Intracellular protein delivery plays a pivotal role in advancing biomedicine and biotechnology, with the potential to revolutionize therapeutic strategies, enhance research capabilities, and open up novel applications [1, 2, 3]. This technology allows researchers to investigate cellular processes and disease mechanisms with greater precision, which is essential for advancing our understanding of complex conditions such as cancer, neurodegenerative disorders, and metabolic diseases [2, 4, 5]. Moreover, it facilitates the screening and development of new drugs by enabling the delivery and evaluation of therapeutic proteins, offering valuable insights into their effects on cellular function and disease pathways. Delivering proteins to specific intracellular compartments also enables the creation of innovative research tools and technologies that can manipulate cellular processes, explore protein interactions, and probe biological functions at a deeper level [6]. In addition, intracellular protein delivery supports the development of personalized medicine by enabling therapies that are tailored to specific molecular targets and individual patient needs. As the field continues to evolve, its potential to impact medicine, biotechnology, and our understanding of biological systems remains vast and transformative.

Recent advancements in nanotechnology, bioconjugation chemistry, and genetic engineering have significantly enhanced intracellular protein delivery methods [7, 8, 9]. These innovations aim to optimize delivery efficiency while minimizing cellular toxicity, thereby expanding the therapeutic potential in both medicine and biotechnology. Key stages of intracellular protein delivery include ensuring stable protein binding and encapsulation within nanoparticles, achieving targeted cellular internalization, and facilitating endosomal escape to release the cargo proteins into the cytoplasm or other cellular compartments [8, 10]. Each of these steps is critical in the design and optimization of protein delivery systems. To improve the binding affinity and stability of protein carriers, the protein of interest is typically encapsulated or complexed with nanocarriers through methods such as physical mixing, covalent conjugation, or the incorporation of specific ligands onto the carrier [7]. These strategies promote multiple noncovalent interactions between the protein cargo and the nanocarrier. After complexation, proteins are usually internalized by cells via endocytosis, where they may become trapped in endosomes or lysosomes, rendering them vulnerable to proteolytic degradation if they do not escape efficiently. To address this challenge, pH‐sensitive peptides or polymers can be engineered to trigger the release of proteins from endosomes into the cytoplasm [11, 12]. Additionally, endosome‐disrupting agents, such as fusogenic peptides or viral‐derived proteins (e.g., influenza hemagglutinin), can facilitate endosomal escape [13, 14]. Recently, several visualization techniques have been developed to monitor the endosomal escape process during the intracellular delivery of biomacromolecules, such as nucleic acids, peptides, and proteins [15, 16, 17]. Techniques like fluorescence microscopy, live‐cell imaging, and advanced imaging modalities enable real‐time observation of endosomal escape dynamics, offering valuable insights into the efficiency and mechanisms of intracellular delivery systems. This knowledge is essential for optimizing therapeutic strategies and elucidating the fundamental mechanisms of intracellular cargo delivery. Once proteins successfully escape the endosomes, they are released into the cytoplasm, where they can interact with cellular targets or perform their intended functions. These functions include enzymatic activity, modulation of signaling cascades, regulation of gene expression, or serving structural roles within the cell.

While significant efforts have been made to improve processes such as complexation, internalization, and endosomal escape, developing reliable methods for detecting the release of cargo proteins from carriers remains a major challenge. The most commonly used approaches involve directly measuring protein activity after intracellular delivery. For example, the enzymatic activity of cargo proteins (e.g., β‐galactosidase, luciferase) can be quantified through substrate conversion assays. Alternatively, released cargo proteins can be detected via immunoblotting or ELISA techniques, using specific antibodies targeting the cargo protein. However, these methods have limitations, as proteins, especially those bound through non‐covalent interactions, may retain biological activity even when still associated with the carrier. Non‐covalent interactions typically do not lead to denaturation or only cause minor alterations in the protein's secondary structure. Fluorescence‐based techniques also offer a way to monitor the release of cargo proteins by tracking changes in fluorescence intensity or localization of fluorophores attached to the proteins. These methods often rely on aggregation‐caused quenching (ACQ), which assesses the dissociation of fluorescent proteins from their complexes by measuring the decrease in fluorescence [18, 19]. However, ACQ generally requires the fluorophores to be densely packed within the complex, and in most cases, only a partial decrease in fluorescence is observed upon dissociation. Fluorescence resonance energy transfer (FRET) provides a more precise reflection of the carrier‐cargo binding dynamics compared to changes in fluorescence intensity alone [20, 21]. FRET involves labeling the cargo protein and carrier with donor and acceptor fluorophores, allowing changes in FRET efficiency to be detected upon cargo release. Despite its advantages, FRET‐based detection methods come with challenges. The attachment of fluorescent molecules to both the carrier and protein can alter their physicochemical properties, potentially influencing cellular uptake and intracellular trafficking. For instance, hydrophobic organic dyes commonly used as fluorophores can enhance hydrophobic interactions between the carrier and the cargo protein, causing them to behave differently from their unlabeled counterparts.

**SCHEME 1:**
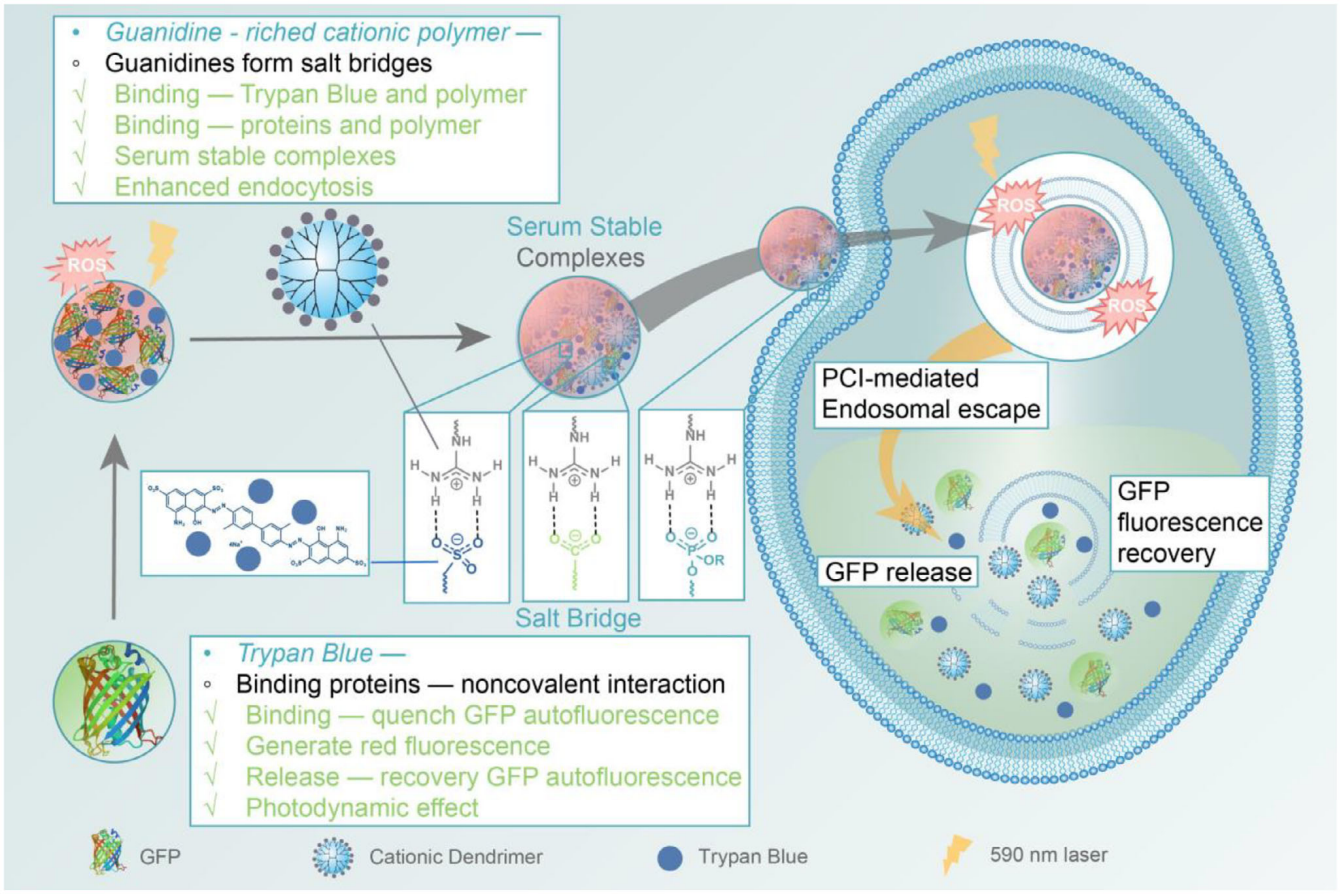
Schematic illustration of the proposed three‐component complexes, designed for serum stability and featuring yellow light‐triggered photodynamic therapy (PCI)‐mediated endosomal escape, facilitating cytosolic protein delivery.

Trypan blue (TB), chemically known as diammonium 3,3'‐([1,1'‐biphenyl]‐4,4'‐diyl)bis(azanediyl)bis(5‐amino‐4‐hydroxynaphthalene‐2,7‐disulfonate), is widely utilized in biomedical research. Its molecular structure allows it to selectively stain dead cells by binding to cytoplasmic proteins after crossing compromised cell membranes [22]. This selective staining is a key component in cell viability assays and other related applications. In addition to its use as a vital dye, TB has been shown to effectively quench autofluorescence emitted by bright green fluorophores [23, 24]. This property makes TB a valuable tool as a cell membrane‐impermeable fluorescence quencher, particularly for suppressing fluorescence from green fluorescent proteins (GFP) and fluorescein‐labeled proteins physically adsorbed to cell membranes [25]. Encouraged by these properties, we sought to explore TB's potential in protein delivery systems as a marker for protein binding and release. TB binds to GFP through supramolecular interactions, resulting in the formation of a negatively charged core. This core then complexes with a cationic polymer rich in guanidine groups to form a nanostructure. Although no green fluorescence was initially observed within the cells, a surprising increase in green fluorescence was detected after irradiation with a 590 nm laser. This unexpected phenomenon, which has not been previously reported, prompts further investigation into the underlying mechanisms. In this study, we demonstrate that the complexation of TB with proteins enhances both its autofluorescence emission and photodynamic effects. These enhancements facilitate endosomal escape through photochemical internalization (PCI), a process crucial for intracellular delivery [26]. Notably, the protein delivery process was traced through the fluorescence quenching and recovery effects of TB, which correlate with protein binding and dissociation, respectively. (Scheme 1)

## 2 Results and Discussion

### 2.1 Characterization of TB/Protein Complexes

**FIGURE 1:**
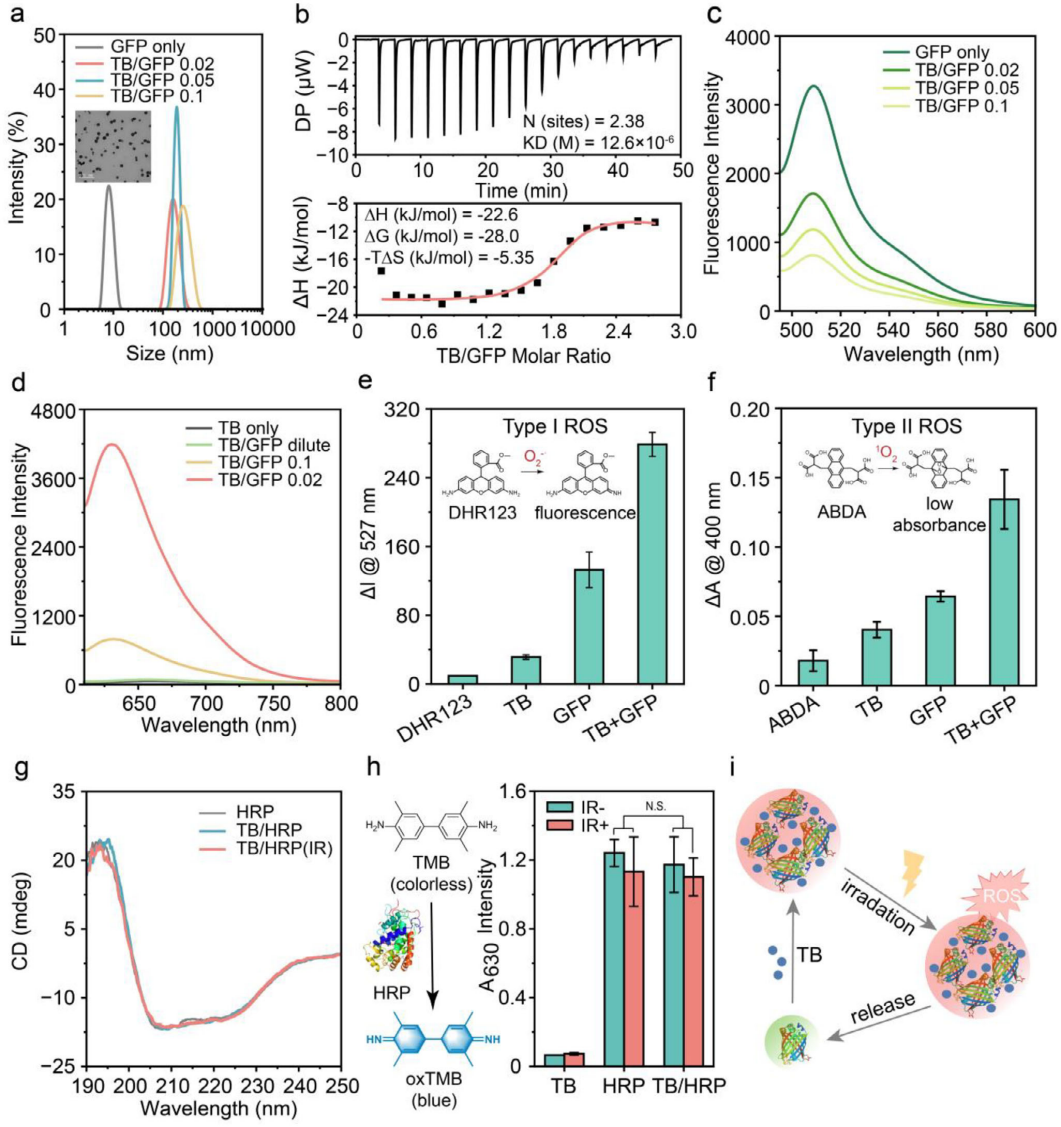
Characterization of TB/protein complexes. (a) Sizes and morphology of TB/GFP complexes at different weight ratios, analyzed by dynamic light scattering (DLS) and transmission electron microscopy (TEM). (b) Isothermal titration calorimetry (ITC) profile for the titration of TB into GFP. (c) Fluorescence spectra of TB/GFP complexes at increasing TB weight ratios. The concentration of GFP was 32 µg/mL. λ_ex_ = 488 nm. (d) Fluorescence spectra of TB/GFP complexes under different conditions. The concentration of TB was 0.8 µg/mL. λ_ex_ = 590 nm. (e) Fluorescence intensity differences at λ_ex_ = 488 nm for DHR123 (10 µm) solution in the presence of different components upon 590 nm light irradiation (22 mW cm^−2^) and the reaction mechanisms of DHR123 for detecting O^2−^ (type I ROS). The concentration of TB and GFP were 0.8 and 32 µg/mL, respectively. (f) Absorbance differences at 400 nm for ABDA (100 µm) in the presence of different components upon yellow light irradiation (22 mW cm^−2^) and the reaction mechanisms of ABDA for detecting singlet oxygen (^1^O_2_, type II ROS). The concentration of TB and GFP were 0.8 and 32 µg/mL, respectively. (g) Effect of TB on GFP's secondary structure, as analyzed by circular dichroism spectroscopy. (h) Determination of the effects of TB/HRP complexes and light irradiation on HRP enzyme activity using an enzyme activity assay kit. The concentration of TB and HRP were 0.8 and 32 µg/mL, respectively (i) Schematic illustration of TB binding with GFP to quench GFP autofluorescence and form red fluorescence complexes, ROS generation under yellow light irradiation, and complex disassembly to release GFP and recover GFP autofluorescence. Data are presented as mean ± standard deviation (n = 3). N.S. *p* > 0.05, ^*^ *p* < 0.05, ^**^ *p* < 0.01, ^***^ *p* < 0.001.

The complexation behavior of TB with proteins was first investigated. As shown in Figure 1a and Figure S1, TB formed uniform, negatively charged nanoparticles when complexed with GFP in aqueous solution at various weight ratios. Isothermal titration calorimetry (ITC) results indicate that the binding of TB to GFP is an enthalpy‐driven, exothermic process, with the binding constant quantitatively determined to be 12.6 × 10^−6^ m (Figure 1b). The interaction between TB and proteins is multifaceted, influenced by the structural characteristics of both the dye and the protein. Key forces driving this interaction include hydrophobic interactions, electrostatic attractions, hydrogen bonding, and π‐π stacking. Upon the addition of TB to the GFP solution, a significant decrease in GFP fluorescence intensity was observed, confirming

TB's ability to selectively quench the autofluorescence emitted by GFP (Figure 1c). Interestingly, the complexation of TB with GFP also resulted in the appearance of red fluorescence (Figure 1d), with the red fluorescence intensity increasing as the protein content was gradually elevated. This phenomenon was similarly observed when TB was complexed with non‐fluorescent proteins, such as bovine serum albumin (BSA) and human serum albumin (HSA), both of which also led to the generation of red fluorescence (Figure S2). Upon dilution of the TB/protein complex, the red fluorescence disappeared, suggesting that protein‐induced assembly plays a crucial role in regulating TB's emission behavior (Figure 1d). This behavior is reminiscent of aggregation‐induced emission (AIE) molecules, which exhibit enhanced fluorescence upon aggregation or nanostructure formation [27, 28]. Similar to AIE molecules, individual TB molecules are relatively non‐fluorescent in benign solvents (water) but exhibit strong red fluorescence in non‐benign solvents (methanol) (Figure S3).

Many AIE molecules exhibit photodynamic properties, with their photodynamic effects being stronger in the aggregated state compared to the dispersed state [29, 30]. Based on this, we hypothesize that TB may also exhibit photodynamic effects upon protein assembly. Dihydrorhodamine 123 (DHR123) is a commonly used probe for detecting Type I reactive oxygen species (ROS), particularly superoxide anions (O_2_^−^), while 7‐diethylaminocoumarin‐3‐carboxylic acid (ABDA) is primarily employed to detect singlet oxygen (^1^O_2_), a Type II ROS. As shown in Figure 1e,f, complexation with GFP significantly enhanced the production of both types of reactive oxygen species (ROS) by TB when irradiated with yellow light (590 nm). To evaluate whether the generated ROS could deactivate the bound cargo proteins, potentially compromising intracellular protein delivery, we selected horseradish peroxidase (HRP) as a model protein. HRP catalyzes the oxidation of the colorless substrate 3,3’,5,5’‐tetramethylbenzidine (TMB) to a blue azo compound in the presence of H_2_O_2_. As shown in Figure 1g, the addition of TB to HRP caused minimal changes to the protein's secondary structure, suggesting that the complex formed between TB and HRP is relatively loose. This observation is consistent with the dilution experiment shown in Figure 1d. Consequently, the enzyme's activity remained largely unaffected after complexation, both in the absence and presence of light irradiation (Figure 1h). Taken together, these results suggest that TB can effectively form a complex with proteins, quenching the autofluorescence emitted by bright green fluorophores, while enhancing its own autofluorescence emission and generating a photodynamic effect. Importantly, this process does not compromise the functionality of the bound cargo proteins (Figure 1i).

### 2.2 Characterization of Cationic Polymers/TB/GFP Complexes

**FIGURE 2:**
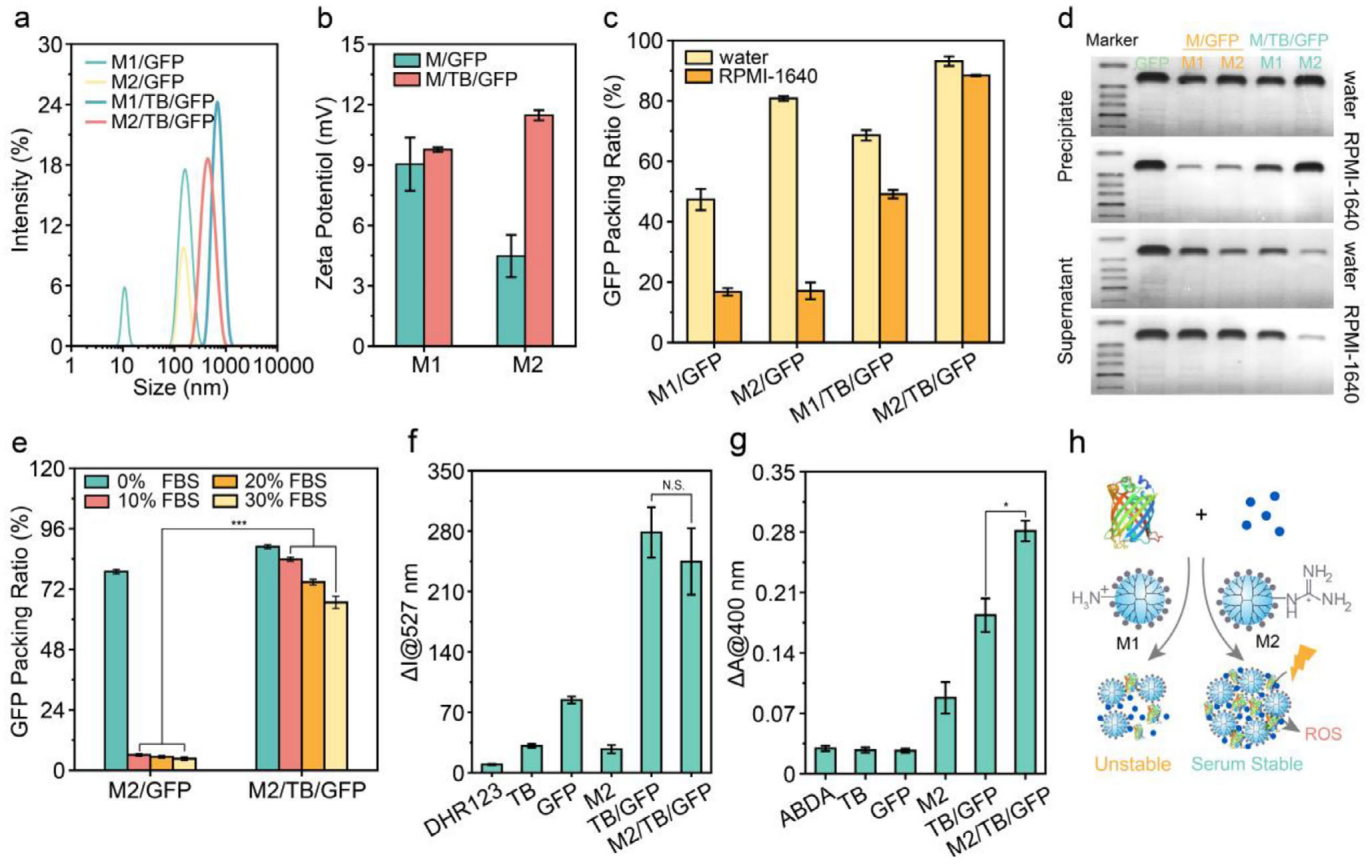
Characterization of cationic polymers/TB/GFP complexes. Size distribution (a) and zeta potential (b) of polymer/GFP and polymer/TB/GFP complexes, analyzed by DLS. (c) Protein packing ratio of polymer/GFP complexes or polymer/TB/GFP complexes in water or RPMI‐1640 medium. The GFP packing ratio was determined by measuring the change in the concentration of free GFP in solution. (d) SDS‐PAGE analysis of polymer/GFP complexes and polymer/TB/GFP complexes. The complexes were prepared in water or RPMI‐1640, followed by centrifugation to separate the precipitate and supernatant. (e) Protein packing ratio of M2/GFP complexes or M2/TB/GFP complexes in cell culture medium with varying fetal bovine serum (FBS) concentrations. The GFP packing ratio was calculated by tracking the concentration change of free GFP in solution. (f) Fluorescence intensity differences at λ_ex_ = 488 nm of DHR123 (10 µm) solution in the presence of different components upon 590 nm light irradiation (22 mW cm^−2^) for the detection of O^2−^. (g) Absorbance differences at 400 nm of ABDA (100 µm) in the presence of different components upon 590 nm light irradiation (22 mW cm^−2^) for the detection of ^1^O_2_. (h) Schematic illustration depicting the stability and ROS‐producing ability of different cationic polymer/TB/GFP complexes under yellow light irradiation. The concentration of TB, GFP, M1, and M2 were 0.8, 32, 24, and 24 µg/mL, respectively. Data are presented as mean ± standard deviation (n = 3). N.S. *p* > 0.05, ^*^ *p* < 0.05, ^**^ *p* < 0.01, ^***^ *p* < 0.001.

After confirming the binding of TB to proteins, we incubated the TB/GFP complex with cationic polymers to prepare transduction complexes. β‐Alanine‐modified (M1) and guanidinopropanoic acid‐modified PAMAM dendrimers (M2) were selected as polymer carriers (Figures S4 and S5). As shown in Figure 2a,b, the guanidine‐rich polymer M2 successfully formed relatively uniform and positively charged nanoparticles with GFP, both in the presence and absence of TB. In contrast, M1 did not form effective complexes with GFP without prior assembly by TB. We also performed TEM experiments. As shown in Figure S6, the morphology of the primary particles, with sizes typically around 100 nm. The discrepancy with the larger DLS measurements is indeed due to aggregation, which is a common phenomenon for such nanoparticles. The TEM images clearly show that this aggregation is more pronounced in the complexes containing TB, providing a direct visual explanation for the DLS data. Furthermore, the inclusion of TB significantly enhanced the protein binding efficiency of both polymers in culture medium (Figure 2c,d), with the M2/TB/GFP complex exhibiting a substantially higher protein binding efficiency (88.3%) compared to M1/TB/GFP (49.5%). Serum stability is a crucial factor when considering the delivery of proteins or protein‐based therapeutics into cells, as serum contains a variety of proteins and factors that can interact with delivery systems. These interactions can lead to the formation of a protein corona or induce nanoparticle aggregation, both of which can significantly affect their stability, cellular uptake, and therapeutic efficacy [31, 32]. As shown in Figure 2e, the addition of serum proteins only slightly reduced the protein encapsulation efficiency of M2 in the presence of TB. In contrast, M2 alone failed to effectively complex with GFP in the presence of serum, underscoring the importance of TB in stabilizing the complex. Given that trypan blue contains sulfonic acid and aromatic groups, the presence of guanidine groups in M2 is expected to enhance the stability of the M2/TB/protein complex through salt bridging and guanidine‐π interactions [33].

We also investigated the photodynamic behavior of the M2/TB/GFP complexes (Figure 2f‐h). As shown in Figure 2f,g, both the binary complexes (TB/GFP, M2/TB) and the ternary complex (M2/TB/GFP) significantly enhanced the production of both type I and type II reactive oxygen species (ROS) compared to TB, M2, or GFP alone. However, the photodynamic effect of TB in the ternary complex was not stronger than in the binary complexes, which may be due to competitive binding between M2, TB, and the protein, potentially altering the binding state of TB within the complex.

### 2.3 Intracellular Delivery Behaviors of Polymer/TB/Protein Complexes

**FIGURE 3:**
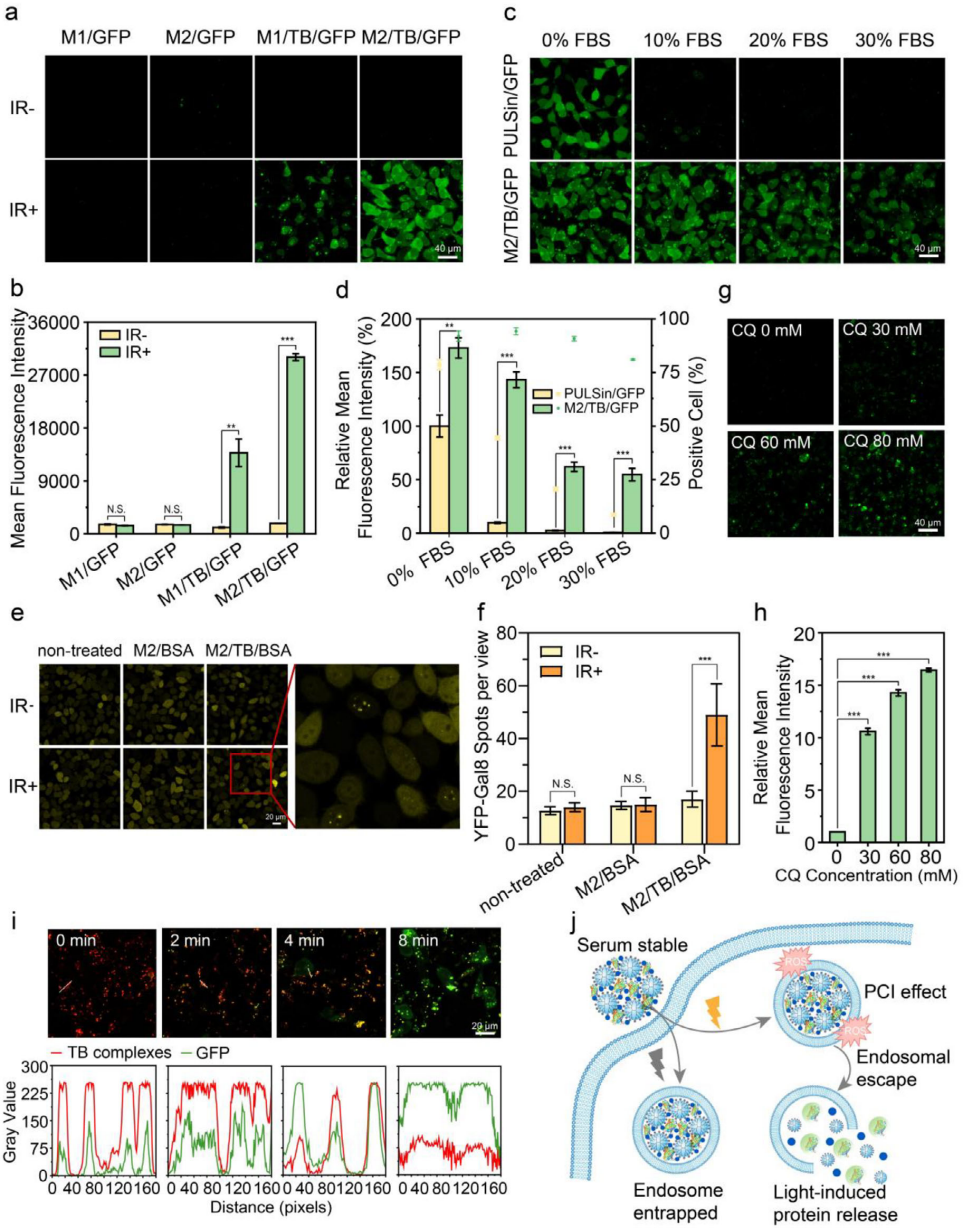
Intracellular characterization of polymer/TB/protein complexes. Confocal images (a) and mean fluorescence intensity (b) of 143B cells treated with polymer/GFP complexes and polymer/TB/GFP complexes, with or without 590 nm light irradiation. Confocal images (c) and mean fluorescence intensity (d) of 143B cells treated with polymer/GFP complexes and polymer/TB/GFP complexes in cell culture medium with varying fetal bovine serum (FBS) concentrations, with or without 590 nm light irradiation. PULSin, a commercial reagent, was used as a positive control according to the manufacturer's protocol. Confocal images (e) and quantitative analysis (f) of aggregated YFP‐Gal8 spots per view in HeLa‐Gal8‐YFP cells treated with M2/BSA or M2/TB/BSA complexes, with or without 590 nm light irradiation. Scale bar, 40 µm. Confocal images (g) and quantitative analysis (h) of 143B cells pretreated with varying concentrations of CQ, followed by treatment with M2/TB/BSA complexes. (i) Confocal images of 143B cells treated with M2/TB/GFP complexes, followed by yellow light irradiation for varying durations. (j) Schematic illustration of photosensitizer‐mediated endosomal escape of M2/TB/protein complexes upon 590 nm light irradiation. The concentration of TB, GFP, M1, and M2 were 0.8, 32, 24, and 24 µg/mL, respectively. Experiments presented were performed under the optimal transfection conditions: an irradiation time of 8 min and a light intensity of 22 mW/cm^2^. Data are presented as mean ± standard deviation (n = 3). N.S. *p* > 0.05, ^*^ *p* < 0.05, ^**^ *p* < 0.01, ^***^ *p* < 0.001.

We then investigated the intracellular protein delivery behavior of the materials. Surprisingly, no fluorescence signal was observed in any of the treated 143B cells (Figure 3a,b). Intracellular protein delivery involves several critical steps, including the formation of stable complexes with the protein cargo, enhanced cellular uptake, endosomal escape, and subsequent cargo release. Given that M2/TB/GFP demonstrated excellent protein binding properties and guanidine‐rich polymers are known for their efficient membrane penetration, we hypothesized that the limited endosomal escape might be a key factor limiting the success of protein delivery with M2. Considering the photodynamic properties of TB upon protein binding, we applied 590 nm yellow laser irradiation to the cells to facilitate successful endosomal escape via the photochemical internalization (PCI) mechanism. As anticipated, irradiation significantly improved the intracellular protein delivery efficiency for both M1/TB/GFP and M2/TB/GFP (Figure 3a,b; Figures S7 and S8). Remarkably, in cells treated with M2/TB/GFP, a large amount of bright green fluorescence was immediately visible after illumination, even under serum‐containing conditions (Figure 3c,d). Importantly, the viability of 143B cells remained unaffected by the irradiation, indicating that the photodynamic behavior of TB promotes endosomal escape without compromising cell viability or proliferation (Figure S9). Moreover, the mechanism study revealed that cellular uptake is significantly impaired by both Wortmannin (an inhibitor of macropinocytosis) and incubation at 4°C (Figure S10). This finding indicates that the complexes are internalized through an active, energy‐dependent pathway, specifically macropinocytosis.

To further visualize the disruption of endosomes under yellow light irradiation, we constructed HeLa cells stably expressing yellow fluorescent protein‐tagged galectin 8 (YFP‐Gal8). Gal8 is a cytosolic protein that can rapidly recognize damaged endosomes by binding to glycosylation moieties on the inner membrane surface [15, 16]. As shown in Figure 3e,f and Figure S11, HeLa cells treated with the M2/TB/BSA complex exhibited minimal Gal8 recruitment under normal conditions. However, after irradiation, numerous punctate fluorescent spots were observed. In contrast, no Gal8 recruitment was detected in cells treated with M2/BSA, regardless of the absence or presence of irradiation. To further investigate the mechanism of endosomal escape, we co‐treated the cells with chloroquine (CQ), a well‐known inhibitor of endosomal acidification. CQ raises the pH within endosomes, disrupting their membrane integrity and facilitating the release of protein cargo into the cytoplasm. Previous studies have shown that this approach can enhance intracellular delivery efficiency by improving endosomal escape [34, 35]. As shown in Figure 3g,h and Figure S12, the addition of CQ gradually increased the intracellular green fluorescence intensity in M2/TB/GFP‐treated cells without irradiation. These findings collectively suggest that the incorporation of TB into the complexes induces a PCI effect, which promotes endosomal escape without affecting cell viability.

The complexation between TB and GFP quenches the intrinsic fluorescence of GFP, while simultaneously inducing red fluorescence from trypan blue. This phenomenon suggests that it could be used to trace the release of proteins within the cell. As shown in Figure 3i, after 6 h of treatment with the M2/TB/GFP complex, only red fluorescence was observed in 143B cells. However, with increasing exposure to 590 nm light, green fluorescence gradually emerged, while red fluorescence decreased, indicating that GFP was effectively released from the complex. These results demonstrate that this system can be utilized to monitor the release of proteins within cells (Figure 3j).

**FIGURE 4:**
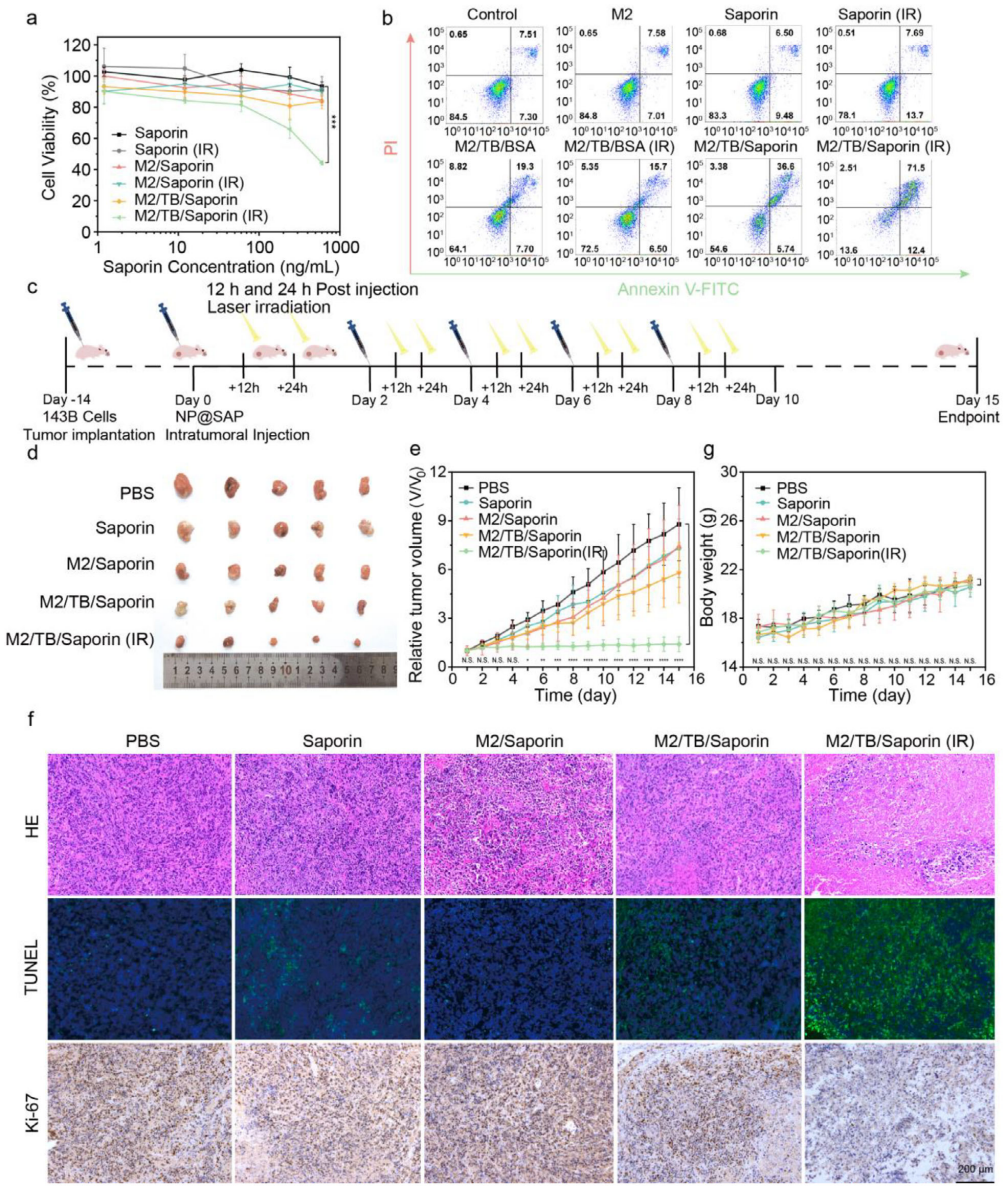
In vitro and in vivo evaluation of M2/TB/Saporin complexes under light irradiation. (a) Cell viability of 143B cells treated with Saporin, M2/Saporin, and M2/TB/Saporin at different Saporin concentrations, with or without light irradiation. The concentration of TB and M2 were 0.8 and 24 µg/mL, respectively. Data are presented as mean ± standard deviation (n = 3). Statistical significance was determined using Student's t‐test at the concentration of (M2/TB/Saporin + IR vs. all other groups). ^*^ *p* < 0.05, ^**^ *p* < 0.01, ^***^ *p* < 0.001. (b) Apoptosis rate of 143B cells treated with M2, Saporin, M2/TB/BSA, and M2/TB/Saporin, under various conditions. The concentration of TB, Saporin, and M2 were 0.8, 32, and 24 µg/mL, respectively. (c) Schematic illustration of the experimental treatment protocols. (d) Representative tumor photographs, (e) relative tumor volumes, and (g) body weight changes of 143B tumor‐bearing mice treated with PBS, Saporin, M2/Saporin, M2/TB/Saporin, and M2/TB/Saporin complexes, with 590 nm light irradiation. Data are presented as mean ± standard deviation (n = 5). Statistical significance was determined using two‐way ANOVA multiple comparison for each time point. N.S. *p* > 0.05, ^*^ *p* < 0.05, ^**^ *p* < 0.01, ^***^ *p* < 0.001. (f) H&E staining, TUNEL staining, and Ki‐67 staining images of tumor sections from each treatment group. Experiments presented were performed under the optimal transfection conditions: an irradiation time of 8 min and a light intensity of 22 mW/cm^2^. Scale bars = 200 µm.

The secondary structure and bioactivity of the cargo protein in the complexes remained unaffected after light exposure (Figure 1g,h), validating the use of this strategy for intracellular delivery of bioactive proteins in tumor therapy. Saporin, a potent ribosome‐inactivating protein (RIP), has gained attention as a potential therapeutic agent for cancer due to its ability to inhibit protein synthesis in target cells, ultimately inducing cell death [36]. As shown in Figure 4a, saporin had minimal effect on the proliferation of 143B cells, likely due to its poor membrane permeability. In contrast, the ternary complex M2/TB/saporin exhibited dose‐dependent antitumor activity upon irradiation, whereas the M2/saporin binary complex did not significantly affect cell proliferation within the tested dose range. Furthermore, irradiation of cells treated with M2/TB/saporin led to a significantly higher apoptosis rate compared to cells treated with M2/TB/BSA, which did not trigger light‐responsive toxicity (Figure 4b). These findings suggest that incorporating TB into the protein delivery system enhances the light‐responsive cytotoxicity of saporin in the treated cells.

### 2.4 In Vivo Protein Delivery Behaviors of M2/TB/Saporin Complexes

The in vivo antitumor efficacy of saporin‐based complexes was evaluated using the 143B subcutaneous tumor model. Treatment began when the tumor volume reached approximately 100 mm^3^ (Figure 4c). Tumors in the PBS group exhibited rapid growth, with the average tumor volume at the endpoint being 8.8 times larger than at the start of treatment (Figure 4d,e). Tumor growth was modestly inhibited in the saporin and M2/saporin groups, with an average tumor volume increase of 7.3 times. The M2/TB/saporin ternary complex showed slight improvements in efficacy compared to the M2/saporin complex and saporin alone, suggesting that the addition of TB enhances complex stability, retention, or cellular uptake. Notably, tumors in the M2/TB/saporin (IR) group exhibited minimal growth, demonstrating that low‐intensity laser irradiation significantly enhanced the antitumor effect of the ternary complex. Histological analyses, including H&E staining, immunohistochemistry (IHC), and immunofluorescence (IF), further supported these findings (Figure 4f). H&E staining revealed the sparsest nuclear distribution in the M2/TB/saporin (IR) group, while TUNEL staining showed a significantly higher proportion of apoptotic cells in this group compared to others. Additionally, Ki‐67 staining demonstrated marked inhibition of cell proliferation following treatment with the M2/TB/saporin complex combined with laser irradiation. Besides, there were no significant differences in body weight or H&E staining of major organs across the treatment groups (Figure 4g), indicating that the complex did not induce systemic toxicity. Considering that TB‐dependent ROS generation may also contribute to tumor suppression, we performed a control experiment using M2/TB/GFP/(IR). As shown in Figure S13, the results clearly show that this control group exhibited no significant anti‐tumor effect, unlike the M2/TB/Saporin/(IR) group. This demonstrates that under our experimental light conditions, TB‐mediated ROS generation does not contribute measurably to tumor suppression. Therefore, the potent therapeutic outcome is unequivocally attributable to the PCI effect of TB, which enables the intracellular delivery of saporin. These results collectively suggest that incorporating TB into the protein delivery system represents a promising strategy for developing biocompatible, light‐responsive drug delivery systems for anticancer therapy.

## 3 Discussion

While our findings demonstrate the promising dual functionality of trypan blue (TB) for protein delivery and tracking, several aspects warrant further consideration and highlight opportunities for future development.

First, the mechanistic details of the TB‐protein‐polymer supramolecular assembly merit deeper investigation. Although we have characterized the binding constant and ROS generation, the precise nanoscale arrangement of TB within the ternary complex and how this arrangement optimizes both fluorescence quenching and photodynamic efficiency remain to be fully elucidated. Understanding these structure‐function relationships could guide the rational design of next‐generation, TB‐inspired delivery agents with enhanced properties.

A critical question is how this approach compares to established PCI strategies, particularly those using clinically evaluated amphiphilic photosensitizers like fimaporfin, which have their roots in the seminal discoveries by Berg, Høgset, and colleagues that established PCI as a viable technology for macromolecule delivery [37, 38, 39, 40, 41]. Unlike fimaporfin, which is designed solely to mediate endosomal escape, TB serves as both the PCI agent and an intrinsic reporter of cargo release via its reversible fluorescence quenching of GFP. This built‐in monitoring capability provides direct, real‐time insight into delivery efficiency without requiring separate labels or ex vivo assays, which is valuable for mechanistic studies and carrier optimization.

We acknowledge that the practical, translational value of the fluorescence tracking feature is currently more academic than immediately clinical. Its primary utility lies in preclinical research, enabling the visualization of release kinetics and the spatial distribution of delivered proteins, which are crucial for optimizing treatment parameters. However, this capability could inform the development of more effective delivery systems and, in the future, potentially be integrated with imaging modalities to confirm successful delivery during localized interventions.

A significant limitation of the current system is the use of 590 nm light, which has limited tissue penetration depth and is therefore suitable mainly for superficial or accessible tumors (e.g., skin, oral cavity). This constrains its direct application for treating deep‐seated malignancies. Future work should explore coupling the TB tracking mechanism with photosensitizers activated by longer‐wavelength light (e.g., near‐infrared) or with upconversion nanomaterials to enable deeper tissue penetration. Alternatively, our approach could be highly relevant for ex vivo applications, such as the engineering of cell therapies, or for localized treatments facilitated by endoscopic or intraoperative light delivery.

## 4 Conclusion

In conclusion, we have established a proof‐of‐concept that leverages the unexpected photochemical properties of TB to create a “self‐reporting” protein delivery system. The complexation of TB with proteins not only enhances its autofluorescence but also potentiates its photodynamic effects, promoting successful endosomal escape through the PCI mechanism. By leveraging TB's fluorescence quenching and recovery properties, we have demonstrated its capability to effectively trace protein dynamics inside cells. Further investigation into the mechanisms underlying TB‐mediated fluorescence changes and its photochemical activation could offer valuable insights for optimizing intracellular delivery strategies and advancing the development of protein‐based therapeutics.

## Conflicts of Interest

The authors declare no conflicts of interest.

## Supporting information




**Supporting File**: advs74944‐sup‐0001‐SuppMat.docx.

## Data Availability

The data that support the findings of this study are available in the supplementary material of this article.
