# Supplementary material for: Visualization of Endosomal Escape of Intracellularly Delivered Protein With Unexpected Photochemical Internalization of Trypan Blue
Source: Adv Sci (Weinh). 2026 Apr 10;13(32):e16456. doi: 10.1002/advs.202516456 (PMC13252647; doi:10.1002/advs.202516456)
Supplement: Supplementary file 1 — Supporting File: advs74944‐sup‐0001‐SuppMat.docx. [file ADVS-13-e16456-s001.docx]

**Supporting Information**

**Visualization of endosomal escape of intracellularly delivered protein with unexpected photochemical internalization of trypan blue**

*Zhongqi Yao, ^#[a]^ Zhan Shi, ^#[a]^ Peng Hu, ^#[b]^ Changping Wang *^[b]^ and Hui Wang*^[a]^*

^[a]^ School of Emergent Soft Matter, South China University of Technology, Guangzhou, 510640, China

^[b]^ Department of Oncology, Shanghai Ninth People’s Hospital, Shanghai Jiao Tong University School of Medicine, Shanghai, 201999, China

* Corresponding author. E-mail: wangh@scut.edu.cn; wangchp@sjtu.edu.cn

**Experimental Section**

**General**

G4 PAMAM dendrimer was purchased from Dendritech (Midland, USA). Boc-beta-alanine, 3-guanidinopropanoic acid, 1-(3-dimethylaminopropyl)-3-ethylcarbodiimide hydrochloride (EDCi), 1-hydroxybenzotriazole (HOBT), trypan blue (TB), and 2-{[10-(2,2-dicarboxyethyl) anthracen-9-yl] methyl} propanedioic acid (ABDA) were purchased from Bidepharm (Shanghai, China). Methyl 2-(3,6-diamino-9H-xanthen-9-yl) benzoate (DHR123) was obtained from Yuanye Biotech (Shanghai, China). Dimethyl sulfoxide (DMSO), triethylamine (TEA) and trifluoroacetic acid (TFA), were purchased from Adamas (Shanghai, China). PULSin was obtained from Polyplus Transfection (Illkirch, France). Chlorpromazine (CPZ), genistein (GEN), methyl-β-cyclodextrin (m-βCD), wortmannin and Chloroquine (CQ) were purchased from MedChemExpress (Shanghai, China). Annexin V-FITC apoptosis detection kit was purchased from Beyotime (Jiangsu, China).

**Synthesis and characterization of M1 and M2**

Boc-beta-alanine (0.2 mmol) or 3-guanidinopropanoic acid (0.2 mmol) was dissolved in 2 mL anhydrous DMSO and mixed with EDCi (0.22 mmol) and HOBT (0.22 mmol) in 2 mL anhydrous DMSO. Triethylamine (TEA, 0.3 mmol) was then added to the solution. The reaction mixture was stirred for 2 h at room temperature. Afterward, G4 PAMAM dendrimer (dissolved in 1 mL anhydrous DMSO) was added to the solution and stirred at room temperature for 48 h. Upon completion of the reaction, the solution was transferred into a 3500 Da dialysis bag and dialyzed against DMSO three times, followed by dialysis against deionized water five times. The dialysate was then freeze-dried to yield the products as white powders. For M1, the product was re-dissolved in 2 mL trifluoroacetic acid (TFA) and 6 mL DMSO. After stirring at room temperature for 6 h, the solution was purified using the same dialysis process described above. The products were characterized by ¹H NMR spectroscopy (Bruker, 500 MHz, 10 mg/mL in D₂O) to determine the average number of ligands attached to each dendrimer.

**Preparation and characterization of** **TB/GFP, and polymer/TB/GFP complexes**

The TB/GFP complexes were prepared by mixing 8 μg of GFP protein, various amount of Trypan blue (TB), and 20 μL of deionized water. For the polymer/TB/protein complexes, 6 μg of polymer was added to the mixture, and the solution was incubated for 20 minutes. After incubation, the mixture was diluted with 50 μL of serum-free RPMI-1640 medium and incubated for an additional 10 minutes. The complexes were then further diluted to a final volume of 250 μL or 1 mL with deionized water or RPMI-1640 medium containing varying concentrations of FBS (0%-30%) for subsequent experiments.

The size and zeta potential of the formed complexes were characterized by dynamic light scattering (DLS) using a Malvern Zetasizer Nano ZS 90 (Malvern, UK) at 25°C. The morphology of the nanoparticles was observed by transmission electron microscopy (TEM) using a JEM 1400 Plus (JEOL, Japan).

**Protein binding ratio of polymer/protein complexes**

The fluorescence spectra of the complexes were recorded using a fluorescence spectrophotometer (F-320, Guangdong, China). Sodium dodecyl sulfate polyacrylamide gel electrophoresis (SDS-PAGE) was performed to determine the protein binding to the polymers. Briefly, a 12.5% polyacrylamide gel and electrophoresis buffers were prepared according to standard protocols. The complexes were centrifuged at 10,000 rpm for 20 minutes to separate the precipitate and supernatant. The samples were then freeze-dried and re-dissolved in 1x protein loading buffer (Sangon Biotech, China). The solutions were heated at 98°C for 10 minutes, after which they were loaded onto the gel and subjected to electrophoresis at 80 V until the bromophenol blue dye reached the bottom of the gel. A commercial protein marker (Sangon Biotech, China) was used to determine the molecular weight of the proteins. The gel was stained with Coomassie Brilliant Blue G-250 (Sangon Biotech, China) and imaged using a gel imaging system (Gelview 5000 Pro, China).

**Cell culture and cytosolic protein delivery**

143B cells (human osteosarcoma cell line, ATCC) and NIH-3T3 cells (mouse embryo fibroblast cell line, ATCC) were cultured in DMEM (Gibco, USA) supplemented with 10% fetal bovine serum (FBS) (Wisent, China) and 1% penicillin-streptomycin (Gibco, USA) at 37°C in a 5% (v/v) CO_2_ incubator. The cells were seeded into 48-well plates or glass-bottomed confocal dishes for further experimentation.

The complexes were prepared as described previously. Once the cell density reached approximately 80%, the medium was removed, and the cells were washed three times with PBS buffer. The complexes were then added to the 143B cells and incubated for 6 h, after which the complex solution was removed. To quench the autofluorescence of eGFP on the cell membrane and outside the cells, TB (0.2 mg/mL) was applied, and the cells were washed three times with PBS buffer. Light-treated groups were irradiated with LED light at a wavelength of 590 nm for 8 minutes. Fluorescence intensity was observed using a laser scanning confocal microscope (LSCM, LSM880 NLO, Germany) and quantitative analyzed using a flow cytometer (FACS Verse, USA).

**Extracellular reactive oxygen species (ROS) detection**

The preparation of the complex solutions was performed as described previously. DHR123 was added to a final concentration of 10 μM. Fluorescence intensity at 527 nm was measured both before and after 8 minutes of light irradiation. An increase in fluorescence intensity was considered indicative of O₂⁻ generation.

In a separate experiment, after the initial preparation steps, H₂O₂ was added to a final concentration of 200 μM, and ABDA was added to 100 μM. Absorbance at 400 nm was measured before and after 8 minutes of light irradiation. A decrease in absorbance was considered indicative of ¹O₂ generation.

**Gal8 recruitment assay**

M2/TB/BSA complexes were prepared as described previously. NIH-3T3 cells stably expressing YFP-Gal8 were seeded onto glass-bottomed confocal dishes and treated with the complexes. After 6 h, the cells were washed three times with PBS. The cells were then irradiated with LED light (590 nm) for 8 minutes. The aggregation of YFP yellow fluorescence in the cells was observed and imaged using a confocal laser microscope. The number and area of yellow fluorescence aggregation points in each group were quantitatively analyzed using ImageJ software.

**Cell viability and apoptosis assay**

143B cells were seeded in 96-well plates and treated with polymer/protein complexes at different concentrations for 6 h, followed by light irradiation for 8 minutes or no irradiation. After treatment, the medium was removed and replaced with fresh medium containing 10% FBS for an additional 18 h. Cell viability was assessed using the CCK-8 assay. Cells without any treatment served as the control group.

For the apoptosis assay, after 6 h of treatment with the protein complexes, 143B cells were stained with propidium iodide and FITC-Annexin V for 15 minutes, following the instructions of the apoptosis staining kit. The stained cells were then analyzed using a flow cytometer.

**Animal experiments**

All animal experiments were conducted in accordance with the guidelines for the care and use of laboratory animals, and were approved by the Ethics Committee of Shanghai General Hospital (2023AW041). Male BALB/c nude mice (approximately 4 weeks old) were purchased from JiHui Laboratory Animal Co., Ltd. (Shanghai, China).

Each 4-week-old healthy nude mouse received a subcutaneous injection of 120 µL of 143B cell suspension (approximately 2 × 10⁶ cells) in the right flank region. After 2 weeks, the tumors reached an average volume of approximately 100 mm³. The mice were then randomly divided into five groups (PBS, saporin, M2/saporin, M2/TB/saporin, M2/TB/saporin (IR); n=5 per group). Mice were intratumorally administered 30 µL of one of the following treatments, according to their group allocation: PBS, PBS-saporin complex (containing 0.6 µg/mL of Saporin), PBS- M2/saporin complex (containing 0.6 µg/mL of saporin and 24 µg/mL of M2), or PBS- M2/TB/saporin complex (containing 0.6 µg/mL of saporin, 24 µg/mL of M2, and 0.8 µg/mL of TB). For the M2/TB/saporin (IR) group, tumors were irradiated with a 590 nm laser (energy density approximately 10.56 J/cm²) for 8 minutes per session at 12- and 24-h following drug injection. This treatment protocol was repeated five times.

Tumor dimensions and body weight were monitored daily. When the maximal tumor volume reached approximately 1500 mm³, all mice were euthanized. Tumor tissues were then collected and sectioned for histological analysis, including hematoxylin-eosin (H&E), Ki-67, and TUNEL staining. Major organs were also collected, sectioned, and stained with H&E to assess potential systemic toxicity. Tumor growth was quantified using the following equation:

Relative tumor volume rate (day n) = tumor volume (day n) / tumor volume (day 0)

**Statistical analysis**

The above assays were at least three independent times. The speciﬁcs of the statistical tests used for each experiment and the number of repetitions are shown in the ﬁgure legends. Significance analysis was performed using either Student’s t-test or GraphPad with a two-tailed test, as detailed in the corresponding notes.

**Figure S1.** Zeta potential of TB/GFP at different weight ratios. The concentration of GFP was 32 μg/mL.


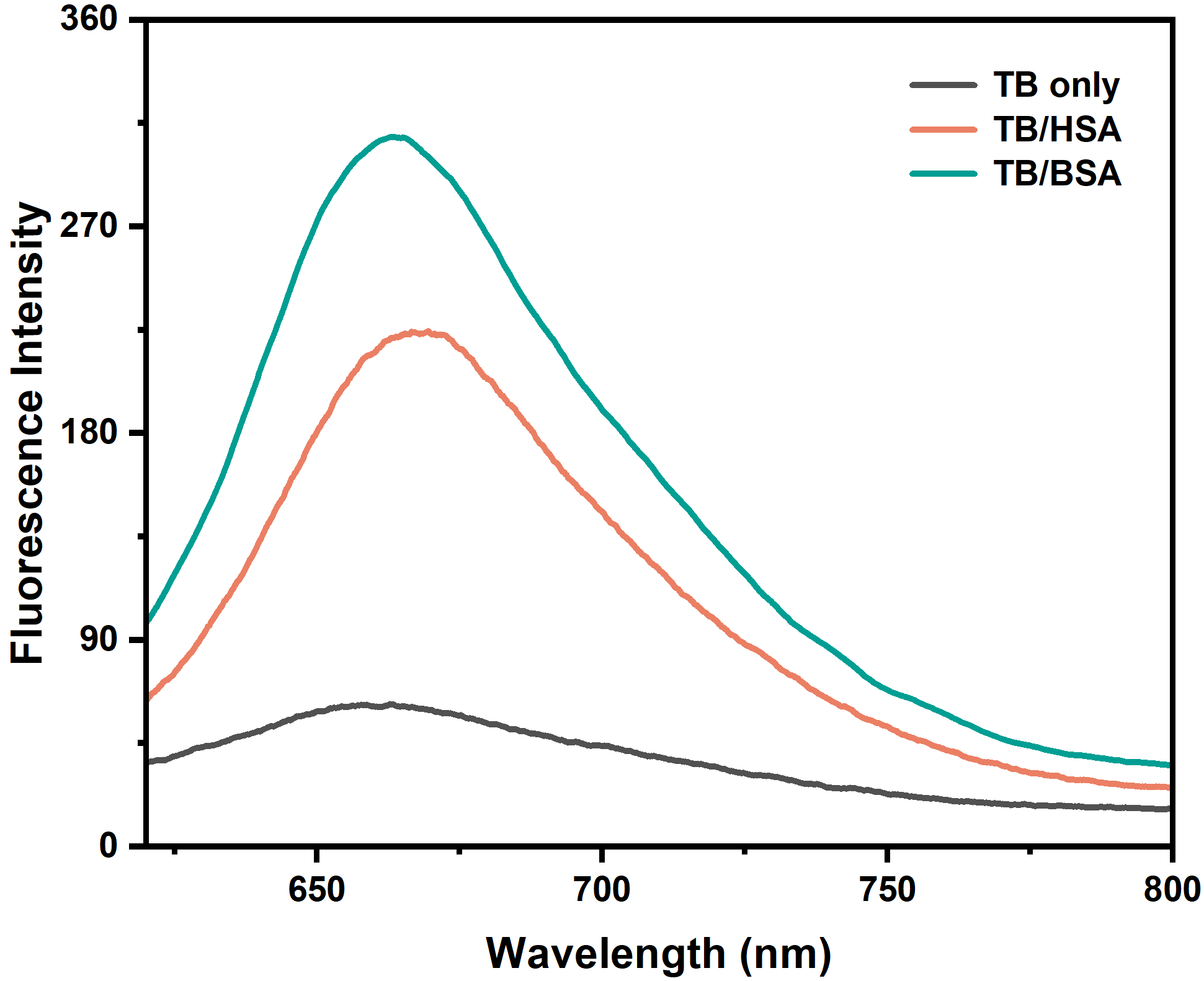


**Figure S2.** Fluorescence spectra of TB/HSA and TB/BSA. λex = 590 nm.

**Figure S3.** Fluorescence spectra of TB in different solvents. λ_ex_ = 590 nm.


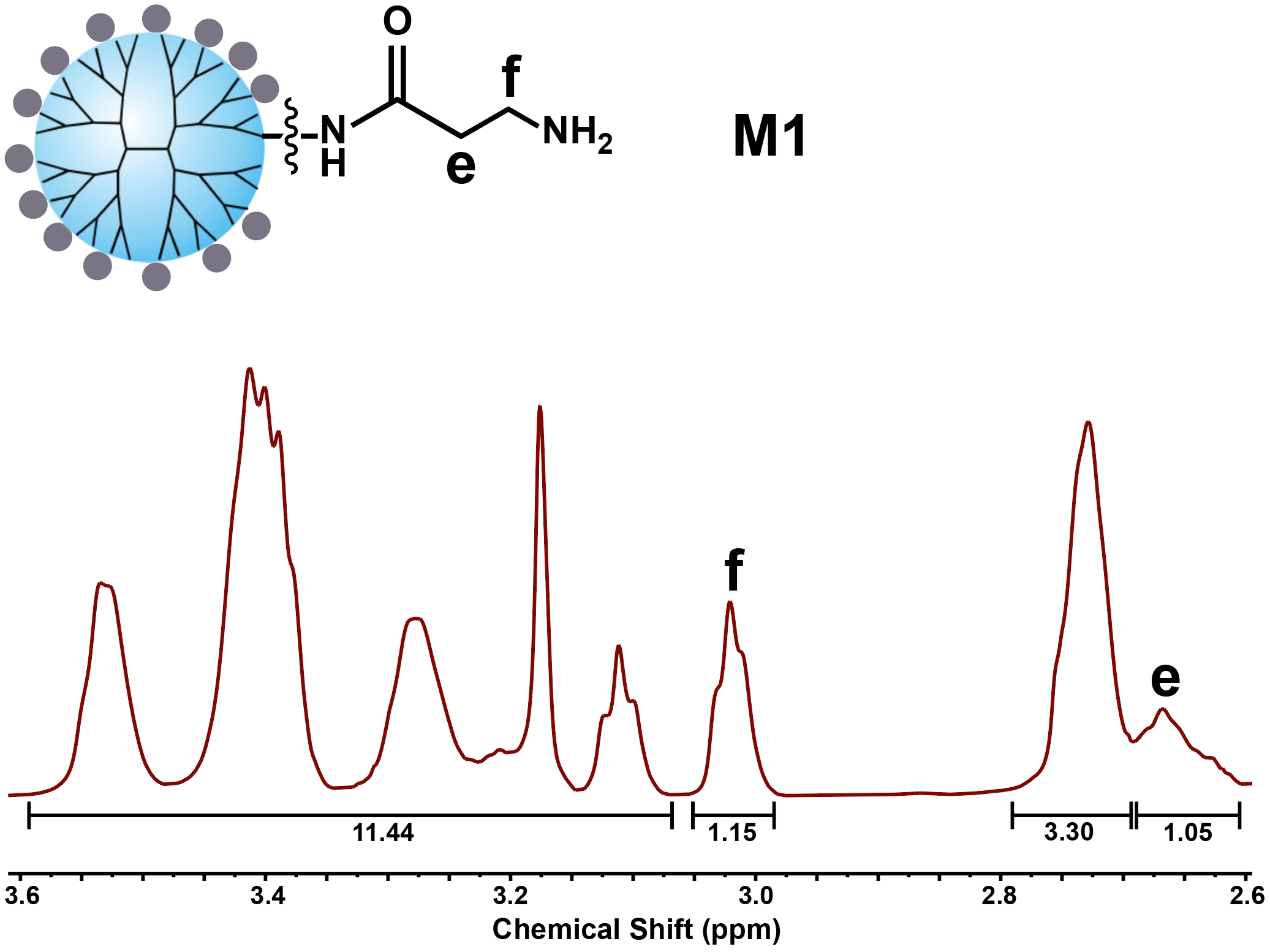


**Figure S4.** ^1^H NMR spectra of M1 in D_2_O.


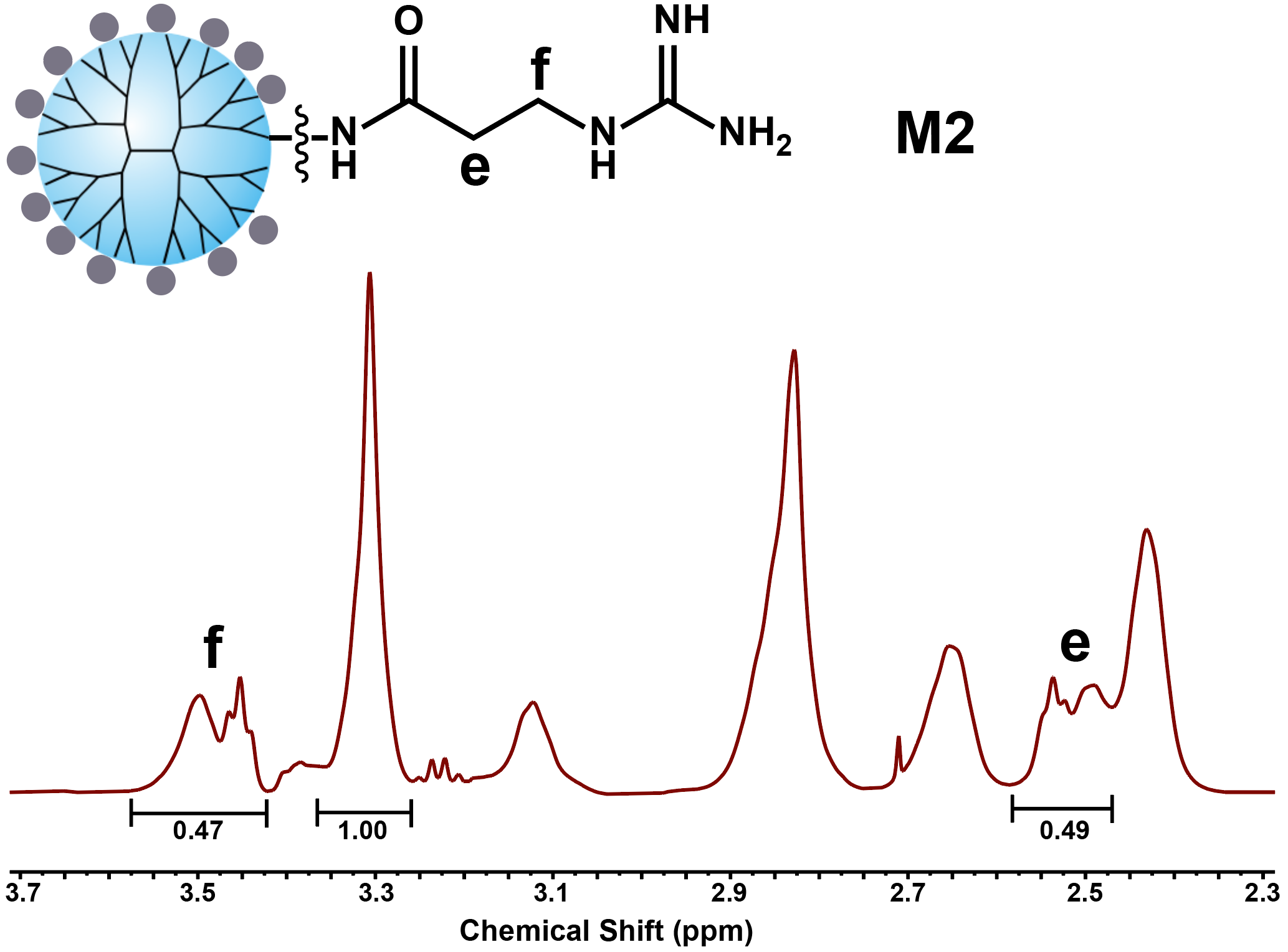


**Figure S5.** ^1^H NMR spectra of M2 in D_2_O.


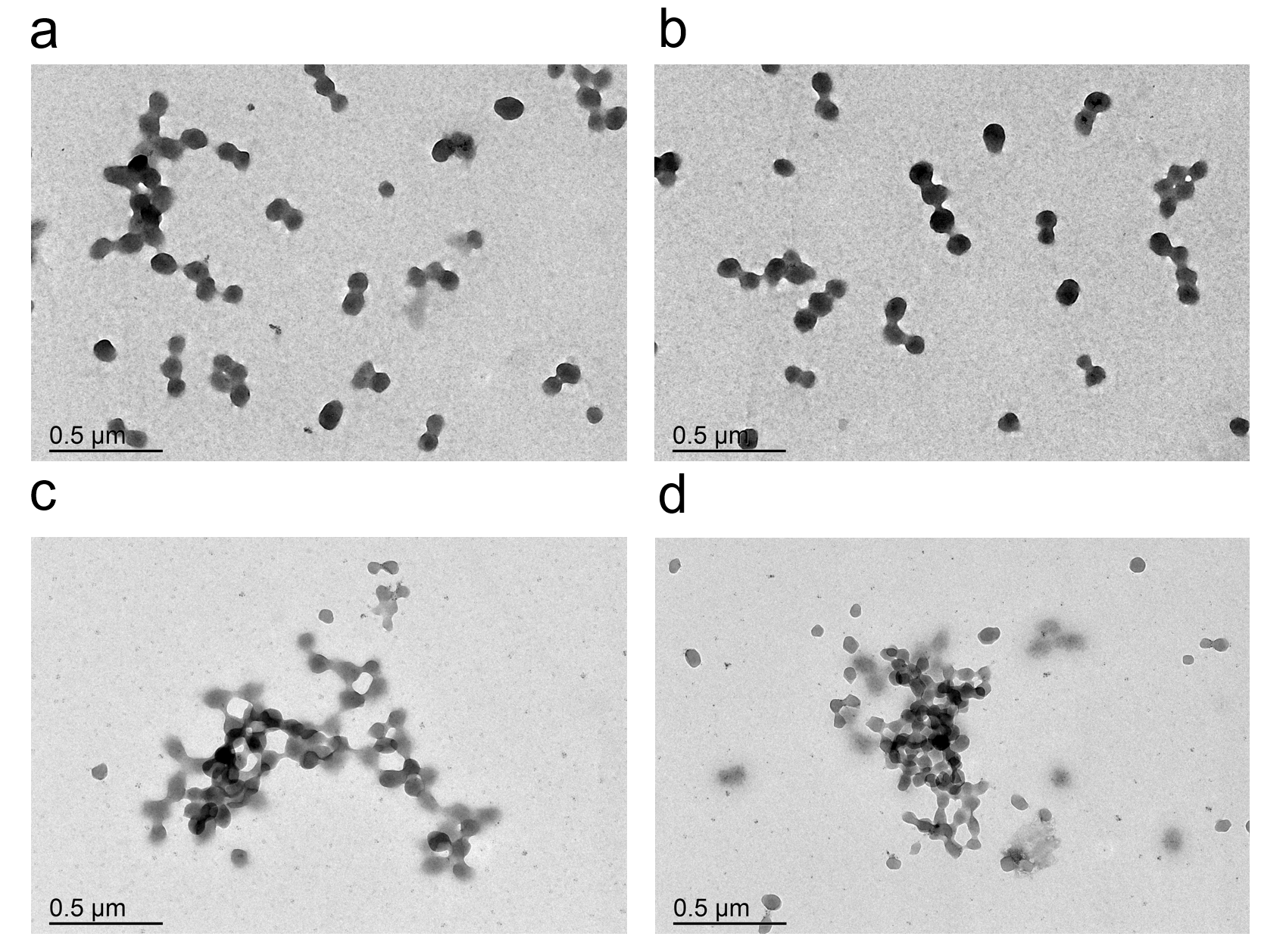


**Figure S6.** Morphology of (a) M1/GFP, (b) M2/GFP, (c) M1/TB/GFP and (d) M2/TB/GFP complexes filmed by transmission electron microscopy (TEM). The concentration of polymer, TB and GFP were 24, 0.8 and 32 μg/mL, respectively. Scale bar: 0.5 μm.


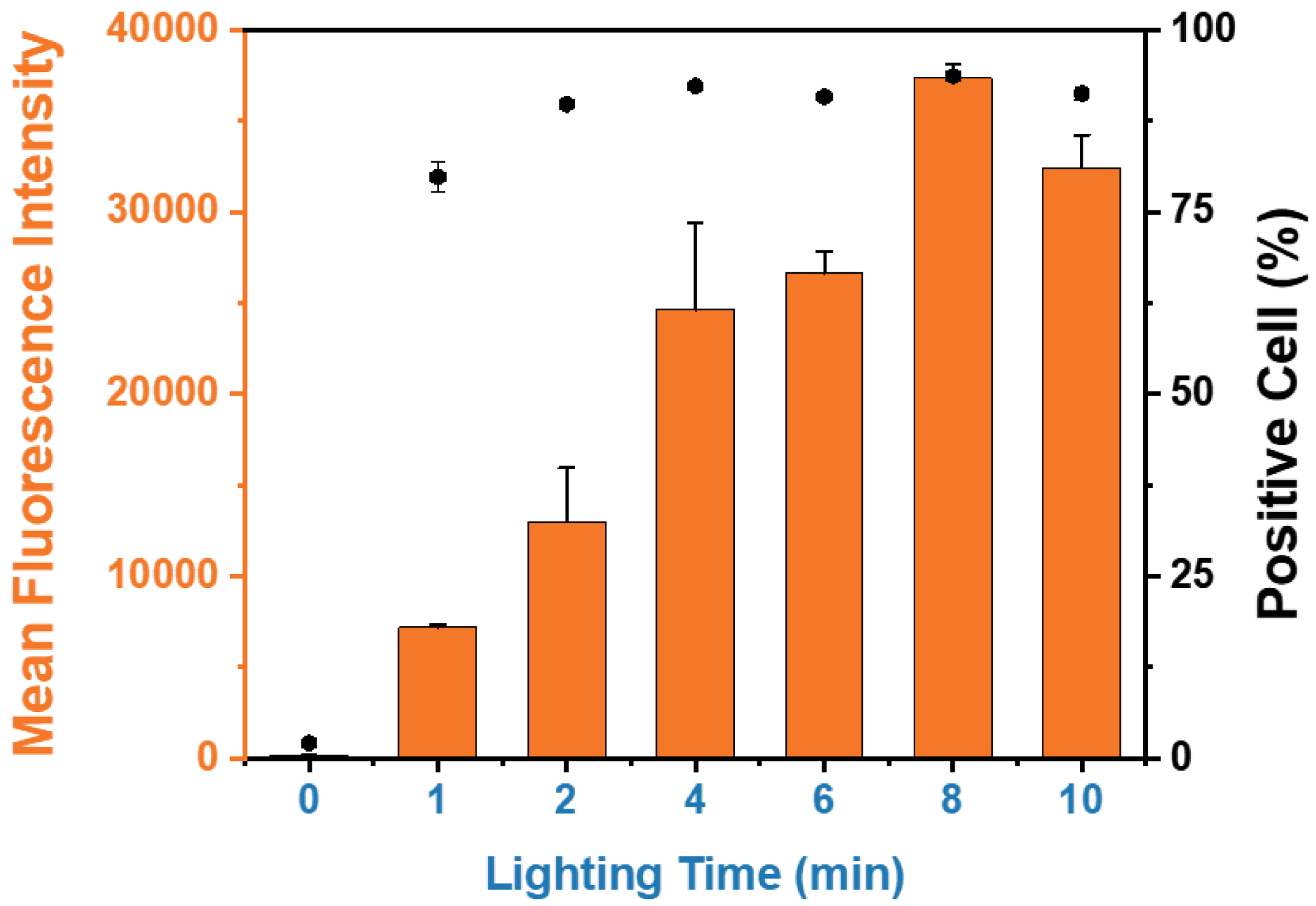


**Figure S7.** Mean fluorescence intensity and positive ratio of 143B cells treated with TB/M2/GFP complexes under different light irradiation times. The concentrations of TB, M2, and GFP were 0.8 μg/mL, 24 μg/mL, and 32 μg/mL, respectively.


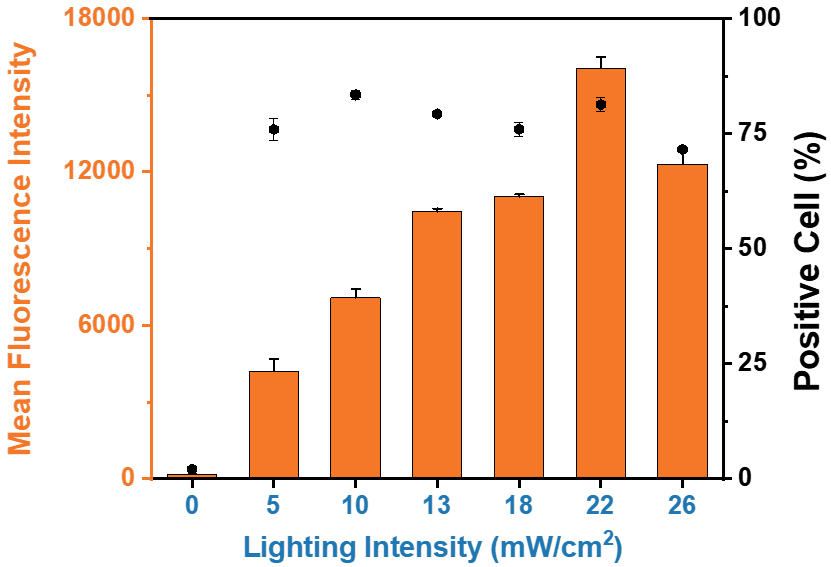


**Figure S8.** Mean fluorescence intensity and positive ratio of 143B cells treated with TB/M2/GFP complexes under different light irradiating intensity. The concentrations of TB, M2, and GFP were 0.8 μg/mL, 24 μg/mL, and 32 μg/mL, respectively.


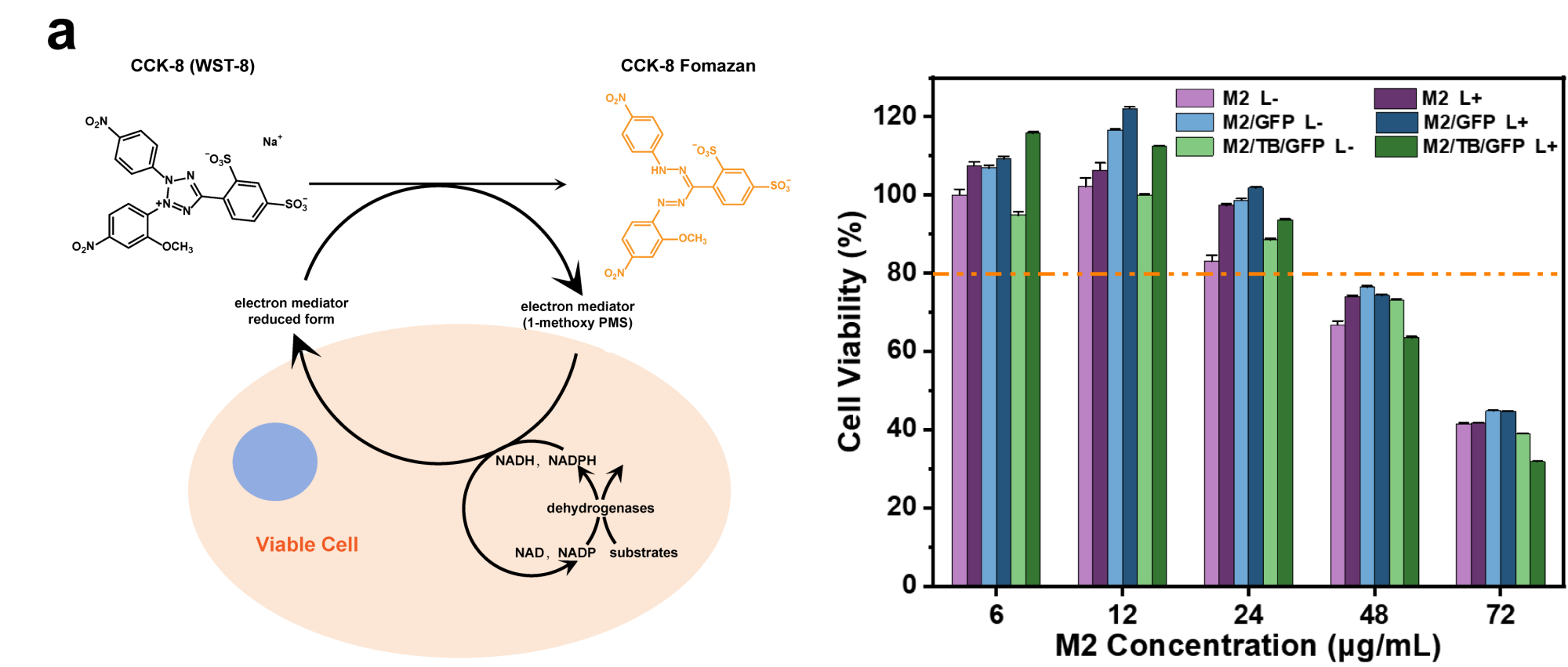


**Figure S9.** Viability of 143B cells treated with different conditions. The concentrations of TB and GFP were 0.8 μg/mL and 24 μg/mL, respectively.


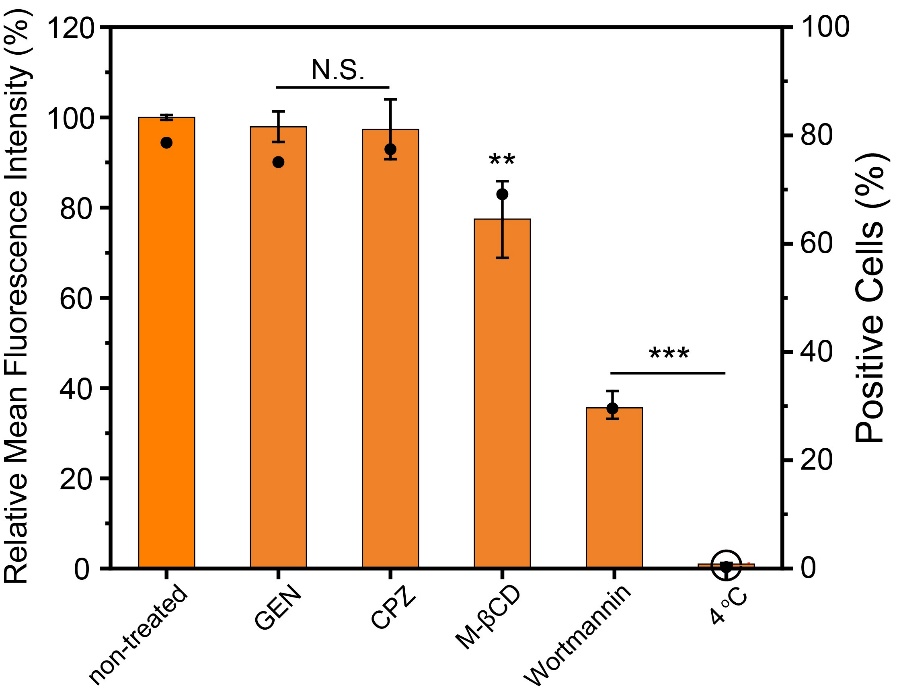


**Figure S10.** Investigation of endocytosis pathway of M2/TB/GFP in 143B cells. The cells were pre-treated with various endocytosis inhibitors, including Genistein (GEN), Chlorpromazine (CPZ), methyl-β-cyclodextrin (M-βCD), Wortmannin for 2 h or 4 ^o^C cultured for 2 h, followed by treatment with the M2/TB/GFP complexes for 6 h and yellow light irradiation for 8 min. The concentration of polymer, TB and GFP were 24, 0.8 and 32 μg/mL, respectively. Data was shown as mean ± s.d. (n=3). N.S. p>0.05, *p<0.05, **p< 0.01, ***p<0.001 were calculated by Graphpad one-way ANOVA.


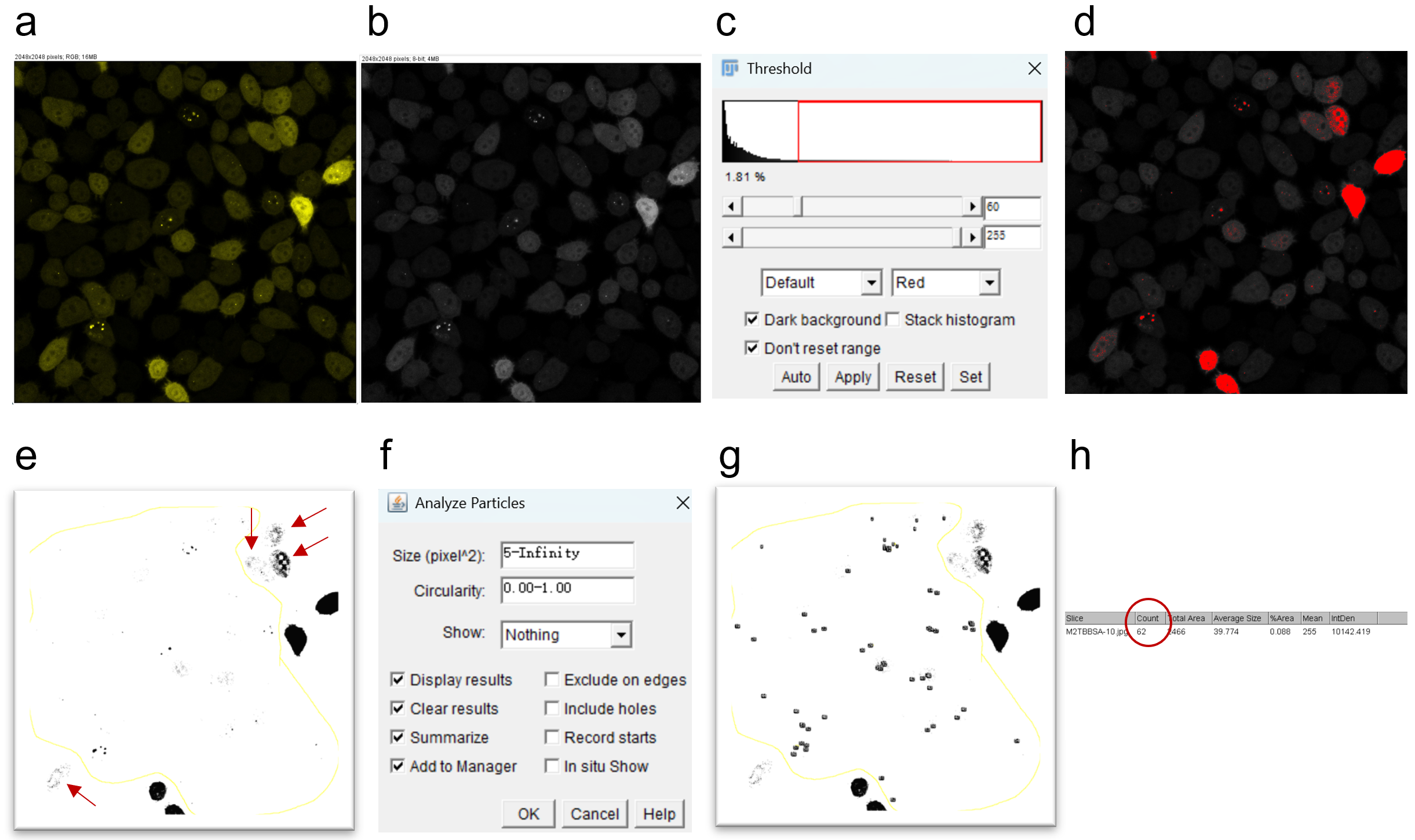


**Figure S11.** Analysis workflow for quantitative analysis of the number of YFP-Gal8 recruitment spots per view in YFP-Gal8-Hela cells using ImageJ. (a) Import the image. (b) Set the image type to 8-bit. (c) Adjust the image threshold according to the above parameters. (d) Obtain the image with highlighted spots. (e) Use the “Freehand Selection” tool to manually outline and exclude abnormal bright spots caused by overexposed cells (indicated by red arrows). (f) Quantify the number of bright spots using "Analyze Particles" with the above parameters. (g) Automatically generate numbers for the counted bright spots. (h) Retrieve the count of bright spots from the generated result window.


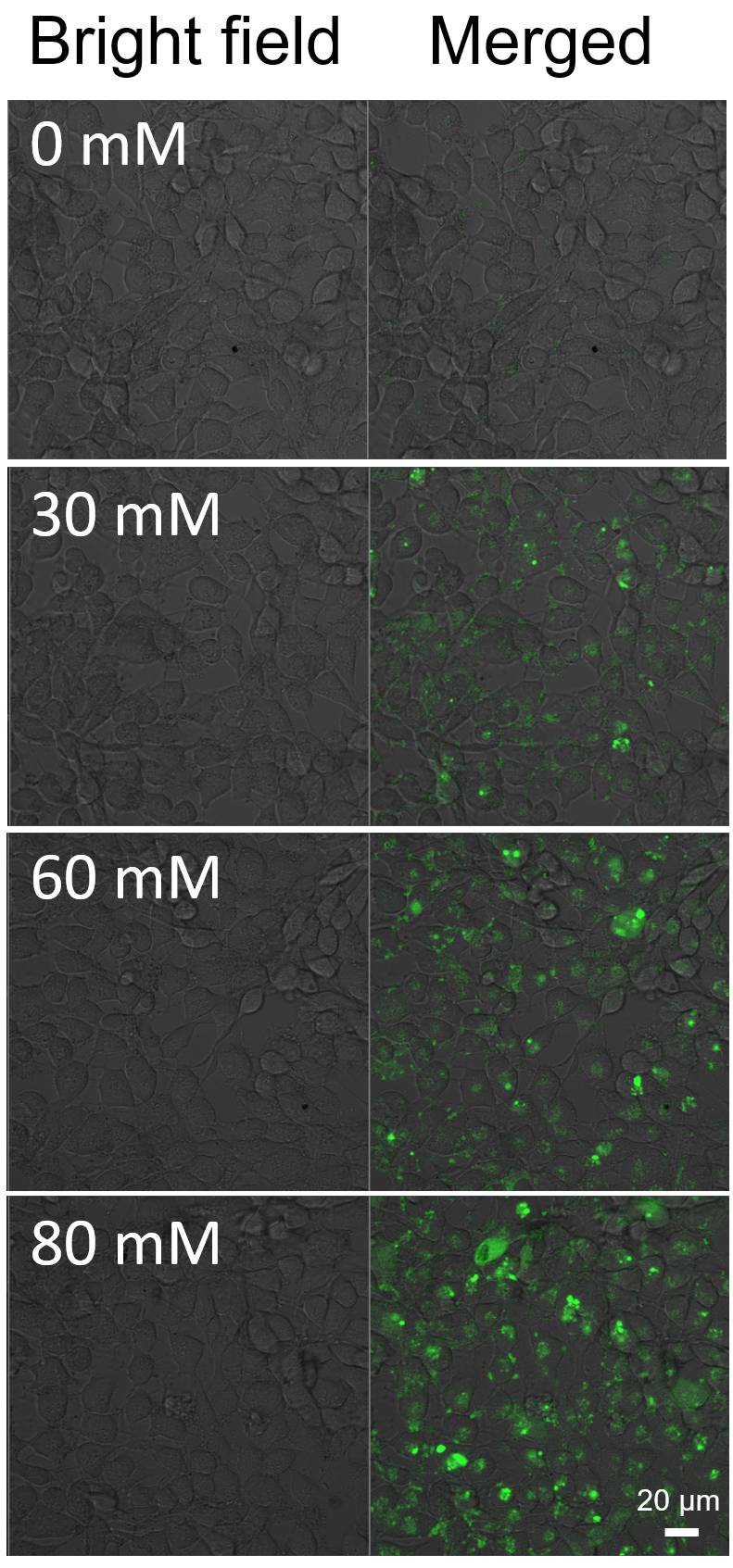


**Figure S12.** Confocal laser scanning microscopy (CLSM) images of 143B cells. The cells were pretreated with 0-80 mM CQ for 2 h, followed by treatment with M2/TB/GFP complexes. The concentration of polymer, TB and GFP were 24, 0.8 and 32 μg/mL, respectively. Scale bar:20 μm.


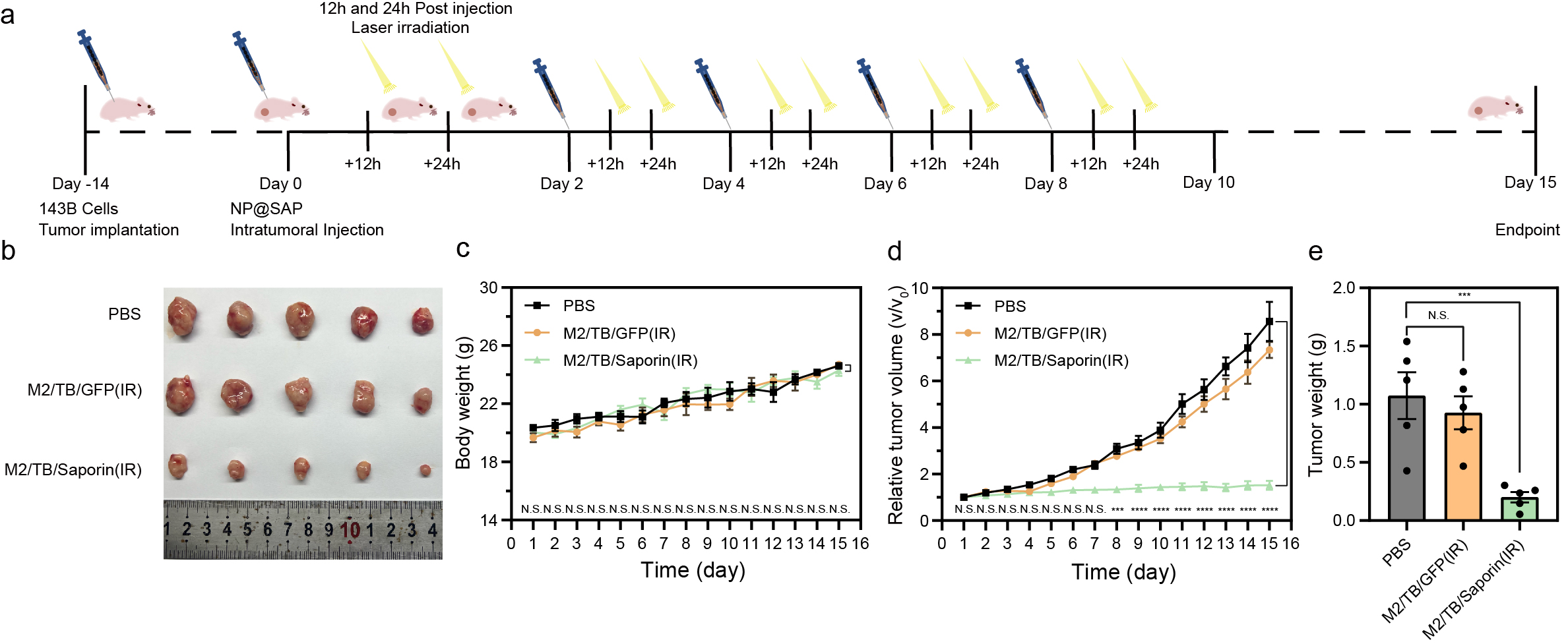


**Figure S13.** In vivo evaluation of M2/TB/GFP and M2/TB/Saporin complexes under light irradiation. (a) Schematic illustration of the experimental treatment protocols. (b) Representative tumor photographs, (c) body weight, (d) relative tumor volumes and (e) tumor weight changes of 143B tumor-bearing mice treated with PBS, M2/TB/GFP, and M2/TB/Saporin complexes, with 590 nm light irradiation. Data was shown as mean ± s.d. (n=5). N.S. p>0.05, *p<0.05, **p< 0.01, ***p<0.001 were calculated by Graphpad two-way ANOVA.


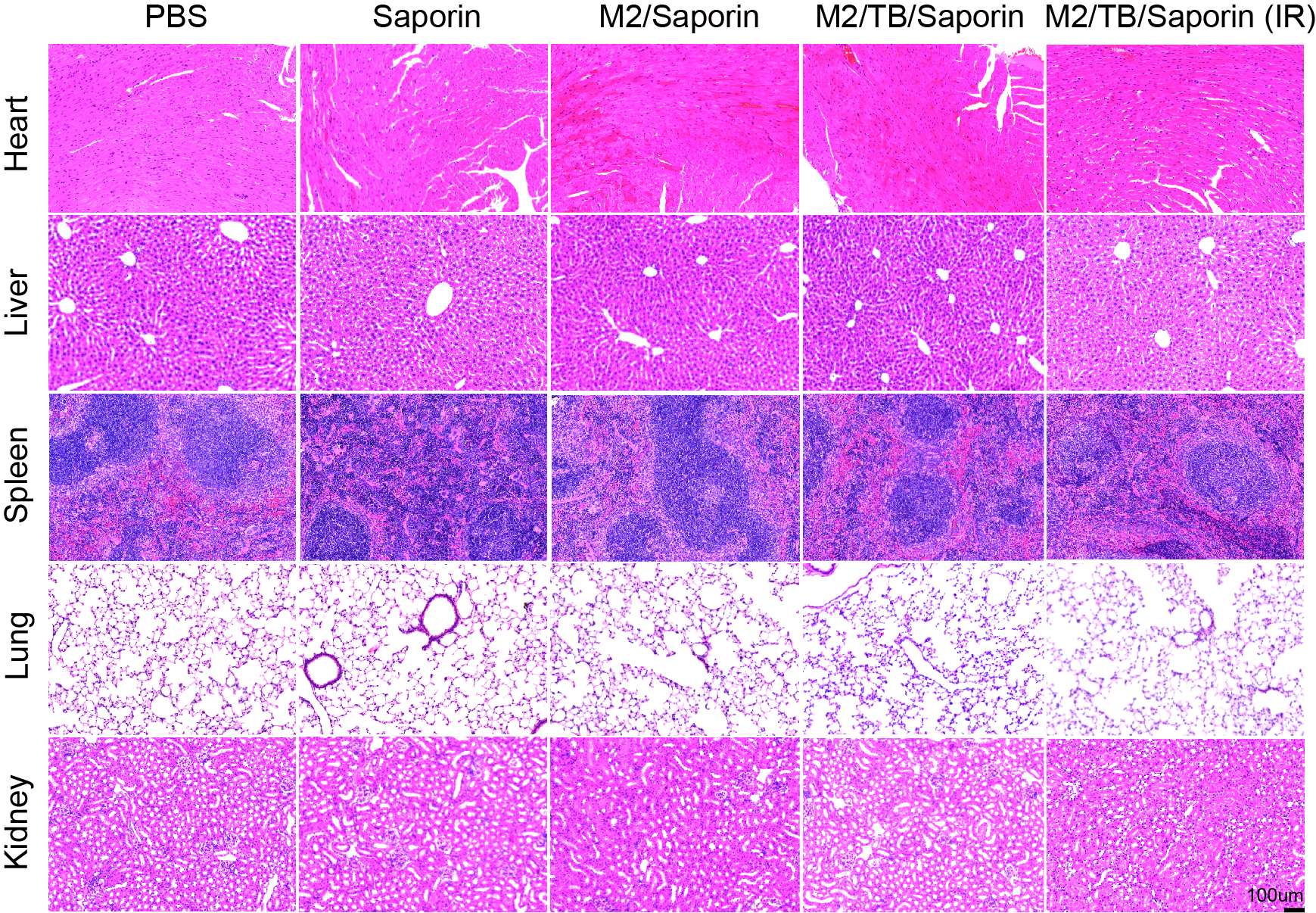


**Figure S14.** H&E staining of different organs from each treatment group.
